# Early prediction of post-stroke depression using polysomnography parameters: a prospective cohort study

**DOI:** 10.3389/fnins.2026.1926299

**Published:** 2026-09-08

**Authors:** Lan Chen, Bin Wang, Dan Kuang, Lei Chen

**Affiliations:** 1 Department of Child Psychiatry, Shaoxing Seventh People’s Hospital, Affiliated Mental Health Center, Shaoxing University, Shaoxing, Zhejiang, China; 2 Department of Neurology, Shaoxing People’s Hospital, The First Affiliated Hospital, Shaoxing University, Shaoxing, Zhejiang, China

**Keywords:** ischemic stroke, machine learning, polysomnography, post-stroke depression, prediction model, sleep disorders

## Abstract

**Background:**

Post-stroke depression (PSD) affects approximately one-third of stroke survivors and significantly impacts functional recovery. This study aimed to develop and validate a prediction model for PSD using polysomnography (PSG) parameters combined with clinical characteristics.

**Methods:**

We conducted a prospective cohort study enrolling 437 acute ischemic stroke patients who underwent PSG assessment within 7 days of stroke onset between January 2022 and December 2024. The primary outcome was PSD at 3-month follow-up, defined as Patient Health Questionnaire-9 (PHQ-9) score ≥10. Multivariate logistic regression identified independent predictors, and machine learning algorithms were compared for model performance.

**Results:**

Among 390 patients completing follow-up, 139 (35.6%) developed PSD. Independent predictors included sleep latency (OR = 2.14, 95% CI: 1.68–2.73), arousal index (OR = 2.13, 95% CI: 1.65–2.94), sleep efficiency (OR = 0.74, 95% CI: 0.55–0.96), heart rate variability RMSSD (OR = 0.77, 95% CI: 0.61–0.96), NIHSS score (OR = 1.35, 95% CI: 1.04–1.76), and prior stroke history (OR = 1.35, 95% CI: 1.09–1.71). The gradient boosting model achieved the highest discriminative performance (AUC = 0.763; bootstrap-validated AUC = 0.738, 95% CI: 0.682–0.794). Risk stratification demonstrated a five-fold gradient in PSD rates across probability categories (14.3 to 70.3%).

**Conclusion:**

PSG parameters, particularly sleep efficiency, sleep latency, and arousal index, are significant independent predictors of PSD. Integrating objective sleep assessment into early stroke management may facilitate identification of high-risk patients for targeted preventive interventions.

## Introduction

Post-stroke depression (PSD) stands as one of the most frequently encountered neuropsychiatric sequelae following cerebrovascular events, with epidemiological studies consistently documenting prevalence rates ranging from 25 to 35% within the first year after stroke onset (Hackett and Pickles, 2014; Robinson and Jorge, 2016). A comprehensive systematic review and meta-analysis incorporating 77 observational studies established an overall pooled prevalence of 27% (95% CI: 25–30%), with clinical interview assessments yielding prevalence estimates of 24% and rating scale measurements indicating 29% (Liu et al., 2023). The clinical significance of PSD extends far beyond its psychiatric manifestations, as affected individuals demonstrate substantially impaired functional recovery, elevated mortality risk, diminished quality of life, and increased healthcare resource utilization compared to non-depressed stroke survivors (Towfighi et al., 2017; Ayerbe et al., 2013). Despite these well-documented adverse consequences, PSD frequently escapes clinical detection and remains inadequately treated, attributable in part to the inherent challenges in distinguishing depressive symptomatology from stroke-related neurological deficits and the persistent absence of reliable early predictive markers (Medeiros et al., 2020).

The pathophysiological mechanisms underlying PSD development involve complex, multifaceted interactions between neurobiological substrates and psychosocial determinants. Neuroimaging studies have repeatedly linked lesions of the frontal–subcortical circuits, particularly those involving the prefrontal cortex and basal ganglia, to the emergence of depressive symptoms (Ferro et al., 2016). In a magnetic resonance imaging study of 591 patients with ischemic stroke, Tang and colleagues reported a significant association between frontal–subcortical circuit infarction and subsequent depression, providing neuroanatomical support for the lesion-location hypothesis (Tang et al., 2011). Additionally, accumulating evidence supports the involvement of inflammatory processes mediated by pro-inflammatory cytokines including interleukin-6 and tumor necrosis factor-alpha, dysregulation of the hypothalamic–pituitary–adrenal axis, and disruption of monoaminergic neurotransmitter systems in PSD pathogenesis (Miller et al., 2009). Recent meta-analytic evidence has confirmed elevated serum inflammatory markers in patients developing PSD, reinforcing the neuroinflammation hypothesis (Zhou et al., 2025). Furthermore, autonomic nervous system dysfunction, as reflected by altered heart rate variability parameters, has emerged as a potential mechanistic link between stroke-induced neural damage and subsequent mood disturbance (Sgoifo et al., 2015; Koch et al., 2019).

Sleep disturbances are remarkably prevalent following stroke and have been consistently associated with unfavorable functional outcomes across multiple domains. A comprehensive systematic review examining dynamic prevalence patterns reported that sleep-disordered breathing affects approximately 67% of stroke survivors in the acute phase, with insomnia symptoms affecting up to 57% of patients (Hasan et al., 2021). The bidirectional relationship between sleep disruption and mood disorders has been extensively characterized in the psychiatric literature, with insomnia functioning both as an antecedent risk factor for depression development and as a consequent manifestation of established depressive illness (Fang et al., 2019; Yasugaki et al., 2025). Polysomnography (PSG) provides objective, comprehensive characterization of sleep architecture, respiratory parameters, and autonomic function through heart rate variability (HRV) analysis, offering quantitative metrics that may serve as early biomarkers for depression risk stratification (Hermann and Bassetti, 2016; Mayer-Suess et al., 2024). Sleep parameters including sleep efficiency, sleep latency, and arousal frequency have demonstrated associations with depressive symptomatology in non-stroke populations, though systematic investigation in stroke survivors remains limited (Baglioni et al., 2011; Alvaro et al., 2013). Recent advances in machine learning methodologies have demonstrated considerable promise in developing accurate PSD prediction models, with meta-analytic evidence reporting pooled c-indices ranging from 0.72 to 0.82 across various algorithmic approaches (Wu et al., 2025).

Despite the recognized importance of sleep disturbances in post-stroke outcomes and the established sleep-mood relationship, relatively few investigations have systematically evaluated comprehensive PSG parameters as predictors of PSD development in a prospective design. Previous studies have predominantly utilized self-reported sleep quality measures or focused narrowly on sleep-disordered breathing, potentially overlooking the broader sleep architecture abnormalities that may contribute to depression vulnerability. Furthermore, the integration of autonomic function parameters derived from overnight PSG recordings with traditional clinical predictors in machine learning frameworks represents an underexplored avenue for enhancing prediction accuracy. This investigation therefore aimed to: (1) develop and internally validate a comprehensive prediction model for PSD utilizing PSG parameters combined with clinical characteristics; (2) evaluate the relative importance of different sleep architecture and autonomic parameters in predicting PSD; and (3) establish risk stratification tools that may facilitate clinical decision-making regarding targeted preventive interventions.

## Methods

### Study design and participants

This prospective cohort study was conducted at a comprehensive stroke center between January 2022 and December 2024. The study protocol received approval from the Institutional Review Board, and written informed consent was obtained from all participants or their legally authorized representatives prior to enrollment.

Inclusion criteria encompassed: age 18 years or older, first-ever or recurrent acute ischemic stroke confirmed by magnetic resonance imaging or computed tomography within 7 days of symptom onset, and capacity to undergo overnight attended PSG. Exclusion criteria comprised hemorrhagic stroke, pre-existing psychiatric disorders requiring ongoing pharmacological management, severe aphasia or cognitive impairment precluding reliable depression assessment utilizing standardized instruments, unstable medical conditions deemed incompatible with overnight monitoring, and current use of sedative-hypnotic medications that might confound sleep architecture assessment.

### Clinical assessment

Comprehensive baseline clinical data were systematically collected within 48 h of hospital admission, encompassing demographic characteristics, stroke severity quantified by the National Institutes of Health Stroke Scale (NIHSS) at admission and again at discharge, stroke subtype classified according to the Trial of Org 10,172 in Acute Stroke Treatment (TOAST) criteria (Adams et al., 1993), and lesion location classified by hemisphere involvement and anatomical region. Pre-stroke and discharge functional status were graded with the modified Rankin Scale (mRS) (van Swieten et al., 1988). Acute reperfusion therapy—intravenous thrombolysis, mechanical thrombectomy, or both—was documented for every patient and delivered in accordance with contemporary guidelines (Powers et al., 2019). All enrolled patients underwent brain magnetic resonance imaging including diffusion-weighted imaging (DWI) within 72 h of admission, and non-contrast computed tomography was performed at presentation for triage and to exclude hemorrhage; infarct volume was quantified on DWI sequences for the entire cohort using semi-automated planimetry, and patients in whom DWI could not be obtained were not enrolled. Medical comorbidities documented included hypertension, diabetes mellitus, dyslipidemia, coronary artery disease, atrial fibrillation, prior stroke history, and current smoking status. Concomitant use of antidepressant, hypnotic, and mood-stabilizing medication was recorded at baseline and at each follow-up contact. Baseline depressive symptoms were assessed using the Patient Health Questionnaire-9 (PHQ-9), a validated instrument with scores ranging from 0 to 27, where higher scores indicate greater depressive symptom severity (Kroenke et al., 2001).

### Polysomnography

Full attended overnight PSG was performed within 7 days of stroke onset using a standardized protocol in a dedicated sleep laboratory environment, with a 32-channel Nihon Kohden Neurofax polysomnography system (Nihon Kohden, Tokyo, Japan). The recording montage included electroencephalography (F3-M2, F4-M1, C3-M2, C4-M1, O1-M2, O2-M1), bilateral electrooculography, chin electromyography, electrocardiography, respiratory inductance plethysmography for chest and abdominal excursion, nasal pressure transducer, oronasal thermistor, pulse oximetry, and bilateral anterior tibialis electromyography. Sleep stages were scored in 30-s epochs according to American Academy of Sleep Medicine criteria by experienced polysomnographic technologists who remained blinded to clinical outcomes (Berry et al., 2012). Each recording was scored independently by two registered technologists; epoch-by-epoch agreement was high (Cohen’s *κ* = 0.86), and discrepant or ambiguous epochs were adjudicated to consensus by a board-certified sleep physician. Derived parameters included total sleep time, sleep efficiency (percentage of time asleep relative to time in bed), sleep latency, REM latency, wake after sleep onset (WASO), percentages of N1, N2, N3, and REM sleep stages, arousal index, apnea-hypopnea index (AHI), oxygen desaturation index (ODI), mean and minimum oxygen saturation, and time with SpO₂ below 90%. Microstructural sleep features were additionally quantified, comprising rapid-eye-movement density (the number of discrete ocular movements per minute of REM sleep) and the non-REM alpha intrusion index (the percentage of non-REM epochs containing alpha-frequency intrusion) (Palagini et al., 2013; Riemann et al., 2020). Heart rate variability was derived from the single-lead overnight electrocardiogram sampled at 256 Hz. R waves were detected with the Pan–Tompkins algorithm (Pan and Tompkins, 1985) and the resulting R–R series was visually verified; ectopic and artefactual beats were removed and normal-to-normal (N–N) intervals were interpolated when they deviated by more than 20% from the local mean. Time-domain indices—the standard deviation of N–N intervals (SDNN) and the root mean square of successive differences (RMSSD)—were computed over consecutive artefact-free five-minute segments during total sleep time and averaged, in accordance with Task Force standards (Task Force of the European Society of Cardiology and the North American Society of Pacing and Electrophysiology, 1996).

### Outcome assessment

The primary outcome was PSD at 3-month follow-up, operationally defined as PHQ-9 score of 10 or greater, a threshold that has demonstrated good sensitivity and specificity for detecting major depressive disorder in stroke populations (Kroenke et al., 2001; Williams et al., 2005). Follow-up assessments were conducted during outpatient clinic visits or via structured telephone interviews for patients unable to attend in-person evaluations. Outcome assessors remained blinded to baseline PSG results throughout the study period.

### Statistical analysis

Continuous variables were expressed as mean ± standard deviation and compared using independent samples *t*-tests following assessment of normality assumptions. Categorical variables were presented as frequencies and percentages and compared using chi-square tests or Fisher’s exact test as appropriate. Pearson correlation coefficients were calculated to examine interrelationships among PSG parameters and associations with PSD outcome. Multivariate logistic regression analysis was performed to identify independent predictors of PSD following adjustment for potential confounders. Candidate predictors comprised admission and discharge NIHSS, pre-stroke and discharge mRS, receipt of reperfusion therapy, established vascular risk factors, sleep architecture and microstructural parameters, and heart-rate-variability indices. Variable selection was guided by clinical relevance, univariate analysis results (*p* < 0.10), and consideration of the events per variable ratio. With 139 PSD events and 11 predictor variables retained in the final model, the events per variable ratio was 12.6, exceeding the commonly recommended minimum threshold of 10.

Model performance was evaluated using the area under the receiver operating characteristic curve (AUC) and precision-recall curves with average precision calculations. Calibration was assessed using calibration plots comparing predicted probabilities against observed event rates and Brier scores. Decision curve analysis was performed to evaluate clinical utility across varying threshold probabilities. Internal validation was conducted using 1,000-iteration bootstrap resampling to derive optimism-corrected AUC estimates. Four machine learning algorithms were systematically compared: logistic regression, random forest, gradient boosting, and support vector machine. The dataset was partitioned into training (75%) and test (25%) sets using stratified random sampling. Risk stratification was performed using predicted probability categories. All analyses were conducted using Python 3.11 with scikit-learn, scipy, and statsmodels packages. Two-sided *p*-values less than 0.05 were considered statistically significant.

## Results

### Study population

**Figure 1:**
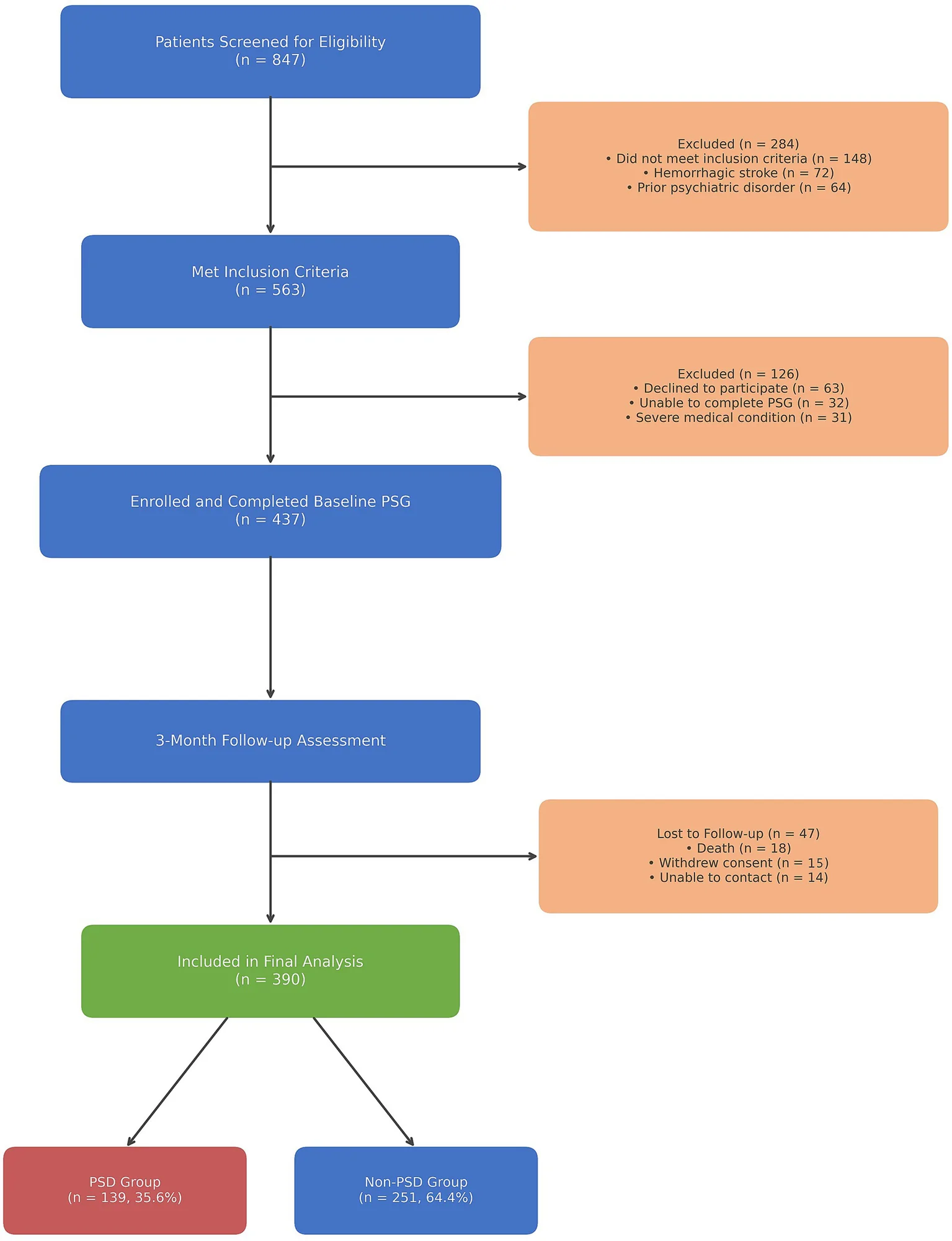
Study flow diagram illustrating patient screening, enrollment, and follow-up procedures. PSD, post-stroke depression; PSG, polysomnography.

Of 847 patients screened for eligibility during the study period, 284 were excluded for not meeting inclusion criteria (*n* = 148), hemorrhagic stroke (*n* = 72), or documented prior psychiatric disorder (*n* = 64). Of the remaining 563 patients meeting inclusion criteria, 126 were further excluded due to declined participation (*n* = 63), inability to complete overnight PSG (*n* = 32), or severe medical conditions precluding study participation (*n* = 31), leaving 437 patients enrolled who completed baseline PSG assessment. At 3-month follow-up, 47 patients (10.8%) were lost to follow-up due to death (*n* = 18), consent withdrawal (*n* = 15), or inability to establish contact (*n* = 14), resulting in 390 patients included in the final analysis. The study flow diagram is presented in Figure 1. Among the 390 patients analyzed, 139 (35.6%) developed PSD at 3-month follow-up, while 251 (64.4%) remained free of clinically significant depression.

### Baseline characteristics

**Figure 2:**
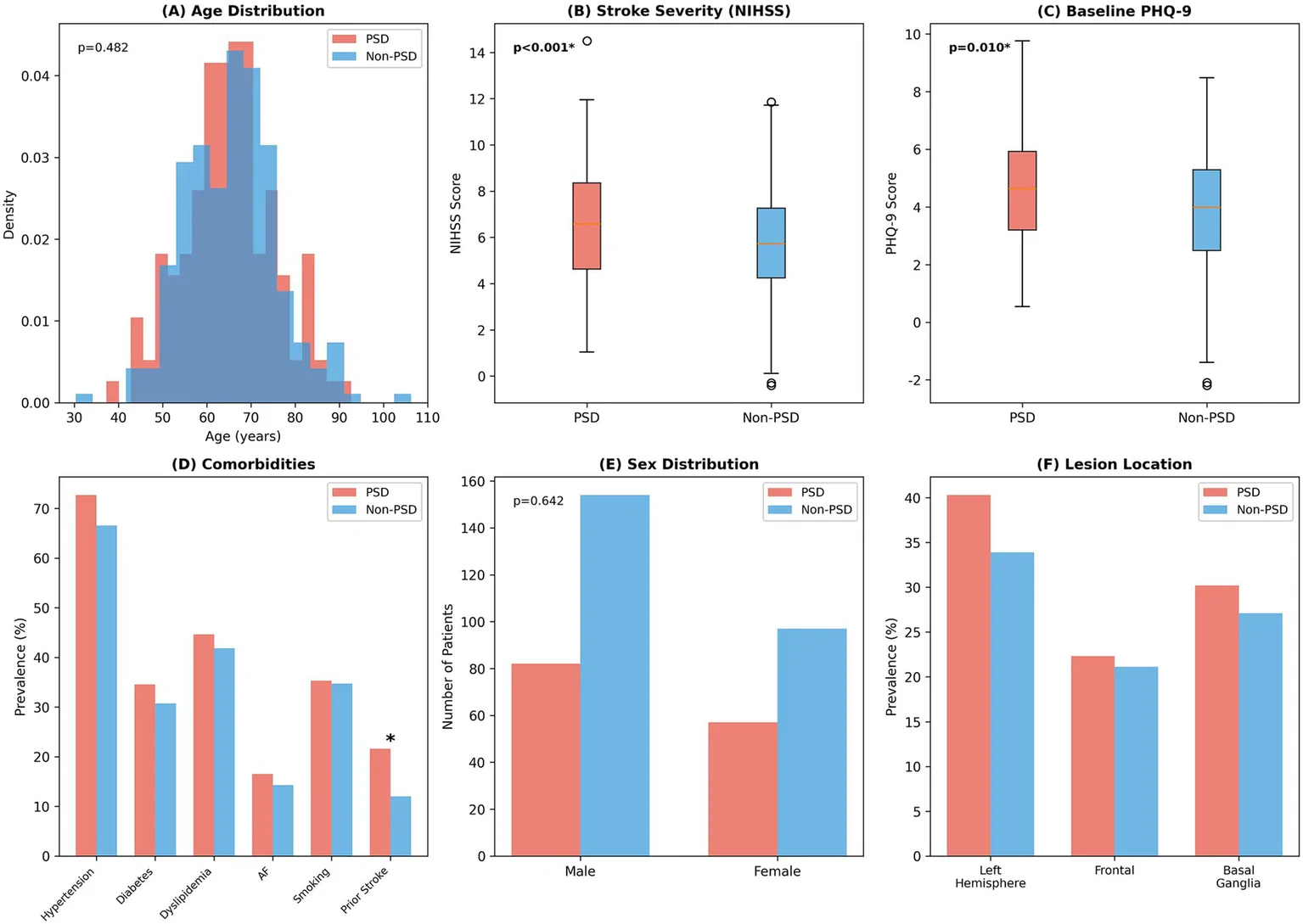
Baseline clinical characteristics stratified by PSD status. **(A)** Age distribution by group. **(B)** Stroke severity assessed by NIHSS scores. **(C)** Baseline PHQ-9 scores. **(D)** Prevalence of major comorbidities. **(E)** Sex distribution. **(F)** Lesion location distribution. PSD, post-stroke depression; NIHSS, National Institutes of Health Stroke Scale; PHQ-9, Patient Health Questionnaire-9. * *p* < 0.05, ** *p* < 0.01, *** *p* < 0.001; PSD group vs. non-PSD group.

Table 1 presents baseline clinical characteristics stratified by PSD status. The mean age of the study cohort was 65.3 ± 10.8 years, with 60.4% male participants. Patients who subsequently developed PSD demonstrated significantly higher NIHSS scores at admission compared to those remaining depression-free (6.8 ± 2.5 vs. 5.8 ± 2.3, *p* < 0.001), indicating greater initial stroke severity in the PSD group. Discharge NIHSS scores were likewise higher in the PSD group (5.9 ± 2.7 vs. 4.6 ± 2.3, *p* < 0.001), and discharge mRS scores indicated greater residual disability (2.7 ± 1.1 vs. 2.2 ± 1.0, *p* < 0.001), whereas pre-stroke mRS did not differ between groups (0.8 ± 0.9 vs. 0.7 ± 0.8, *p* = 0.214). The proportion of patients receiving acute reperfusion therapy was comparable between groups (intravenous thrombolysis 27.3% vs. 29.5%, *p* = 0.646; mechanical thrombectomy 12.9% vs. 11.6%, *p* = 0.699). Baseline PHQ-9 scores, while remaining subclinical in both groups, were marginally elevated in patients who subsequently developed PSD (4.2 ± 2.2 vs. 3.6 ± 2.0, *p* = 0.010). The prevalence of hypertension (72.7% vs. 66.5%, *p* = 0.198) and diabetes mellitus (34.5% vs. 30.7%, *p* = 0.424) did not differ significantly between groups. However, prior stroke history was substantially more common in the PSD group (21.6% vs. 12.0%, *p* = 0.008). Left hemisphere lesion involvement demonstrated a trend toward higher frequency in PSD patients (40.3% vs. 33.9%, *p* = 0.192), though this association did not achieve statistical significance. The distributions of baseline characteristics are illustrated in Figure 2.

**Table 1:** Baseline clinical characteristics by PSD status.

Variable	PSD group (*n* = 139)	Non-PSD group (*n* = 251)	*p* value
Age (years), mean ± SD	65.8 ± 10.9	65.0 ± 10.7	0.482
Male sex, *n* (%)	82 (59.0)	154 (61.4)	0.642
BMI (kg/m^2^), mean ± SD	26.1 ± 4.3	25.6 ± 4.1	0.278
NIHSS score at admission, mean ± SD	6.8 ± 2.5	5.8 ± 2.3	<0.001*
NIHSS score at discharge, mean ± SD	5.9 ± 2.7	4.6 ± 2.3	<0.001*
Pre-stroke mRS, mean ± SD	0.8 ± 0.9	0.7 ± 0.8	0.214
Discharge mRS, mean ± SD	2.7 ± 1.1	2.2 ± 1.0	<0.001*
Intravenous thrombolysis, *n* (%)	38 (27.3)	74 (29.5)	0.646
Mechanical thrombectomy, *n* (%)	18 (12.9)	29 (11.6)	0.699
Baseline PHQ-9, mean ± SD	4.2 ± 2.2	3.6 ± 2.0	0.010*
Hypertension, *n* (%)	101 (72.7)	167 (66.5)	0.198
Diabetes mellitus, *n* (%)	48 (34.5)	77 (30.7)	0.424
Prior stroke, *n* (%)	30 (21.6)	30 (12.0)	0.008*
Left hemisphere lesion, *n* (%)	56 (40.3)	85 (33.9)	0.192

BMI, body mass index; mRS, modified Rankin Scale; NIHSS, National Institutes of Health Stroke Scale; PHQ-9, Patient Health Questionnaire-9; PSD, post-stroke depression; SD, standard deviation. * *p* < 0.05.

### Polysomnography findings

**Figure 3:**
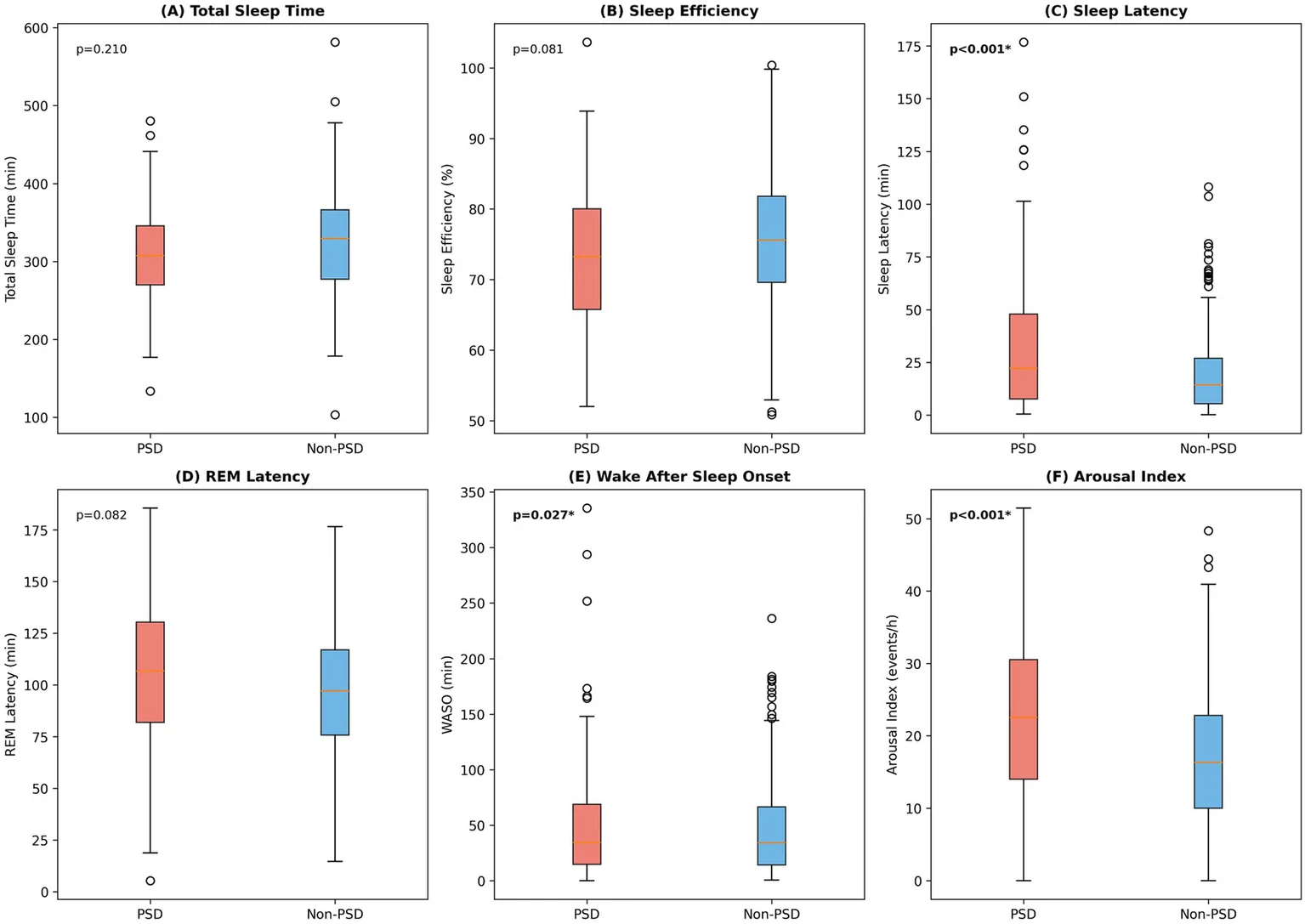
Sleep architecture parameters stratified by PSD status. **(A)** Total sleep time. **(B)** Sleep efficiency. **(C)** Sleep latency. **(D)** REM latency. **(E)** Wake after sleep onset (WASO). **(F)** Arousal index. Box plots display median, interquartile range, and outliers. PSD, post-stroke depression; REM, rapid eye movement. * *p* < 0.05, ** *p* < 0.01, *** *p* < 0.001; PSD group vs. non-PSD group.

**Figure 4:**
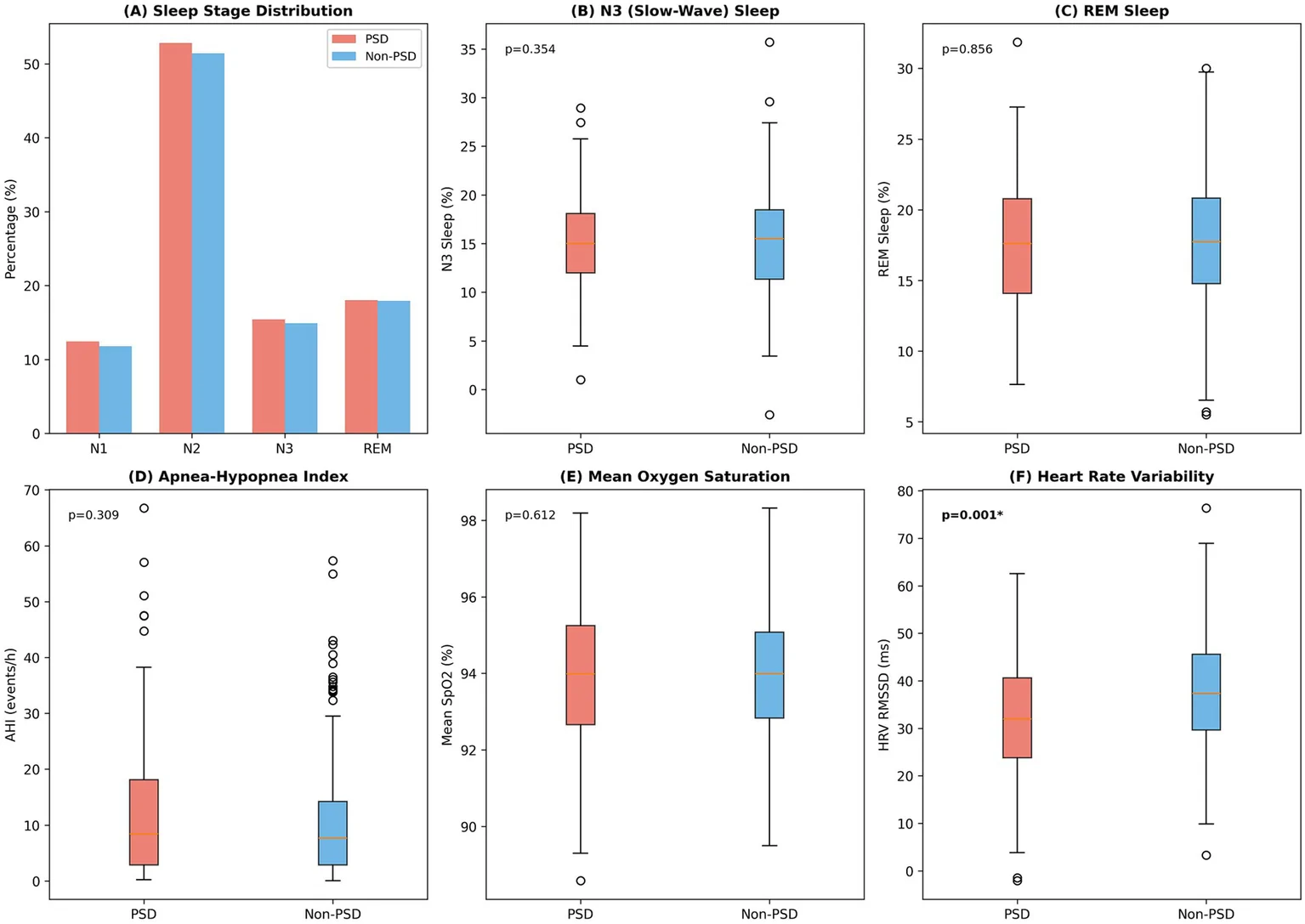
Sleep stages and respiratory parameters stratified by PSD status. **(A)** Sleep stage distribution. **(B)** N3 (slow-wave) sleep percentage. **(C)** REM sleep percentage. **(D)** Apnea-hypopnea index (AHI). **(E)** Mean oxygen saturation. **(F)** Heart rate variability (HRV RMSSD). PSD, post-stroke depression; REM, rapid eye movement. * *p* < 0.05, ** *p* < 0.01, *** *p* < 0.001; PSD group vs. non-PSD group.

PSG parameters comparing the PSD and non-PSD groups are summarized in Table 2 and illustrated in Figures 3, 4. The parameters that reached statistical significance are presented first (Figure 3). Patients who developed PSD exhibited significantly prolonged sleep latency (28.6 ± 20.4 vs. 18.5 ± 12.6 min, *p* < 0.001) and a markedly elevated arousal index (24.1 ± 14.4 vs. 16.3 ± 10.2 events per hour, *p* < 0.001) compared with non-PSD patients. Wake after sleep onset was also significantly greater in the PSD group (52.4 ± 38.6 vs. 43.8 ± 32.4 min, *p* = 0.027).

**Table 2:** Polysomnography parameters by PSD status.

Variable	PSD group (*n* = 139)	Non-PSD group (*n* = 251)	*P* value
Sleep latency (min)	28.6 ± 20.4	18.5 ± 12.6	<0.001*
Arousal index (events/h)	24.1 ± 14.4	16.3 ± 10.2	<0.001*
WASO (min)	52.4 ± 38.6	43.8 ± 32.4	0.027*
Sleep efficiency (%)	74.1 ± 9.6	75.9 ± 9.3	0.081
REM latency (min)	103.2 ± 33.8	95.4 ± 31.2	0.082
Total sleep time (min)	312.5 ± 68.2	321.7 ± 67.4	0.210
REM density (movements/min)	4.8 ± 1.6	4.1 ± 1.5	<0.001*
NREM alpha intrusion index (%)	18.7 ± 7.9	14.6 ± 6.8	<0.001*
AHI (events/h)	10.8 ± 9.7	9.8 ± 9.7	0.309
Mean SpO₂ (%)	93.8 ± 1.8	93.9 ± 1.7	0.612
HRV RMSSD (ms)	32.4 ± 11.8	36.8 ± 12.4	0.001*

AHI, apnea-hypopnea index; HRV RMSSD, heart rate variability root mean square of successive differences; NREM, non-rapid eye movement; REM, rapid eye movement; WASO, wake after sleep onset. Data presented as mean ± SD. * *P* < 0.05.

Two parameters showed a consistent trend toward alteration without reaching statistical significance: sleep efficiency tended to be lower in the PSD group (74.1 ± 9.6% vs. 75.9 ± 9.3%, *p* = 0.081), and REM latency tended to be longer (103.2 ± 33.8 vs. 95.4 ± 31.2 min, *p* = 0.082). Total sleep time did not differ between groups (312.5 ± 68.2 vs. 321.7 ± 67.4 min, *p* = 0.210).

Sleep stage distribution and respiratory parameters are presented in Figure 4. The distribution of sleep stages (Figure 4A) demonstrated similar patterns between groups, with N1 sleep comprising approximately 12%, N2 sleep 52%, N3 sleep 15%, and REM sleep 18% in both groups. Specifically, N3 (slow-wave) sleep percentage did not differ significantly between PSD and non-PSD patients (15.4 ± 5.5% vs. 14.9 ± 5.4%, *p* = 0.354), nor did REM sleep percentage (18.0 ± 4.5% vs. 17.9 ± 4.6%, *p* = 0.856). Regarding respiratory parameters, the apnea-hypopnea index was similar between groups (10.8 ± 9.7 vs. 9.8 ± 9.7 events per hour, *p* = 0.309), as was mean oxygen saturation (93.8 ± 1.8% vs. 93.9 ± 1.7%, *p* = 0.612).

Analysis of microstructural features revealed a higher rapid-eye-movement density (4.8 ± 1.6 vs. 4.1 ± 1.5 movements per minute, *p* < 0.001) and a greater non-REM alpha intrusion index (18.7 ± 7.9% vs. 14.6 ± 6.8%, *p* < 0.001) in patients who developed PSD, indicating that microstructural disruption accompanied the macrostructural sleep changes. Heart rate variability analysis revealed significantly lower HRV RMSSD in the PSD group (32.4 ± 11.8 vs. 36.8 ± 12.4 ms, *p* = 0.001), suggesting reduced parasympathetic tone in patients who subsequently developed depression.

### Correlation analysis

**Figure 5:**
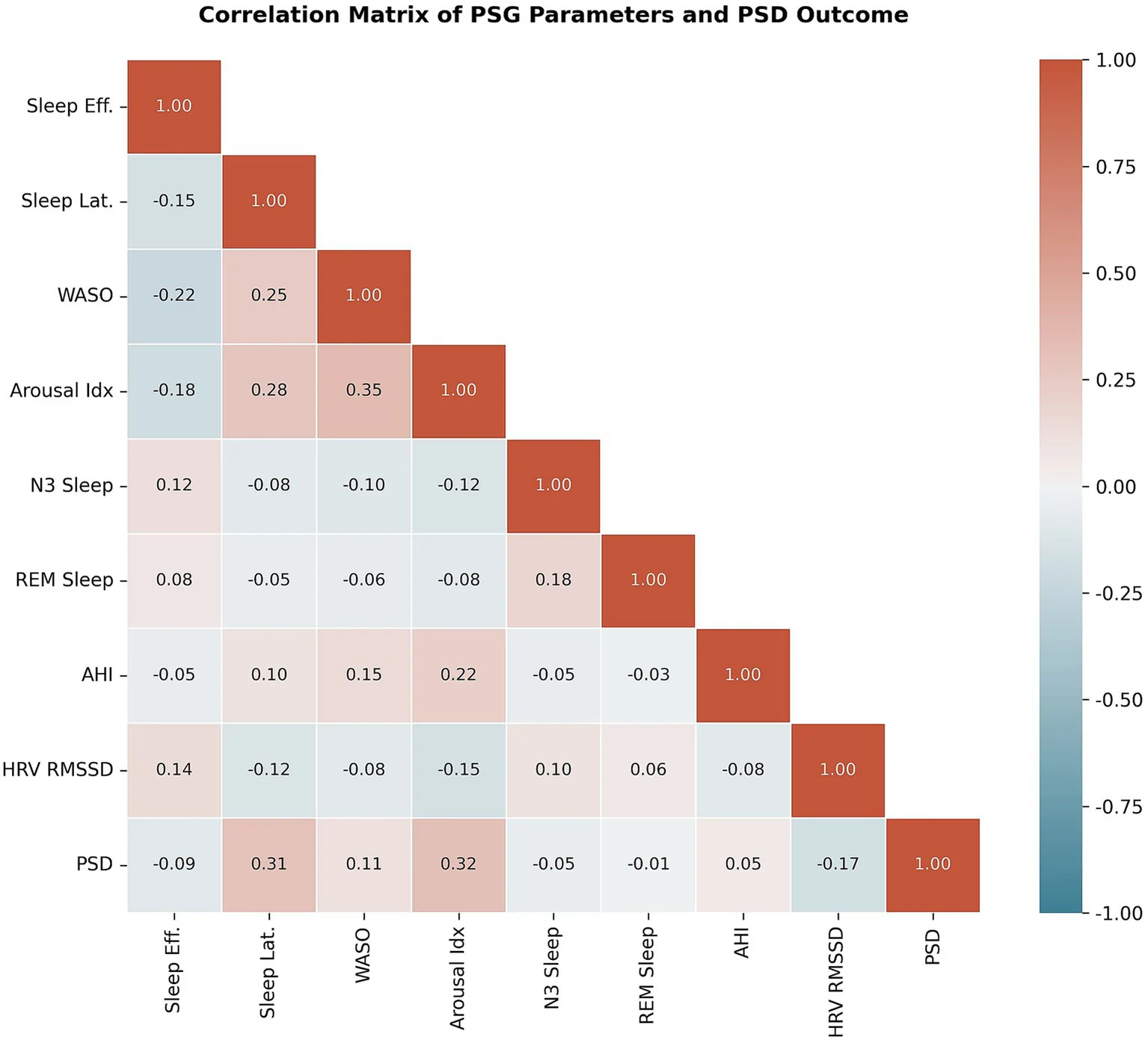
Correlation matrix of PSG parameters and PSD outcome. Color scale indicates Pearson correlation coefficients ranging from −1 (blue) to +1 (red). Lower triangle displays correlation values; diagonal represents perfect self-correlation. PSD, post-stroke depression; WASO, wake after sleep onset; AHI, apnea-hypopnea index; HRV, heart rate variability.

The correlation matrix of PSG parameters and PSD outcome is displayed in Figure 5. Among PSG parameters, sleep efficiency demonstrated weak negative correlations with sleep latency (r = −0.15) and WASO (r = −0.22), consistent with these parameters reflecting related aspects of sleep quality. The arousal index demonstrated positive correlation with PSD outcome (r = 0.32), while sleep latency showed similar positive correlation with PSD (r = 0.31), representing the two strongest correlations between individual PSG parameters and the primary outcome. HRV RMSSD exhibited negative correlation with PSD (r = −0.17), supporting the role of autonomic dysfunction in PSD pathophysiology. Importantly, the modest correlations among predictor variables (all absolute *r* values less than 0.35) indicated acceptable multicollinearity levels. Variance inflation factors for all predictors in the multivariate model ranged from 1.08 to 1.52, confirming low multicollinearity.

### Multivariate logistic regression analysis

**Figure 6:**
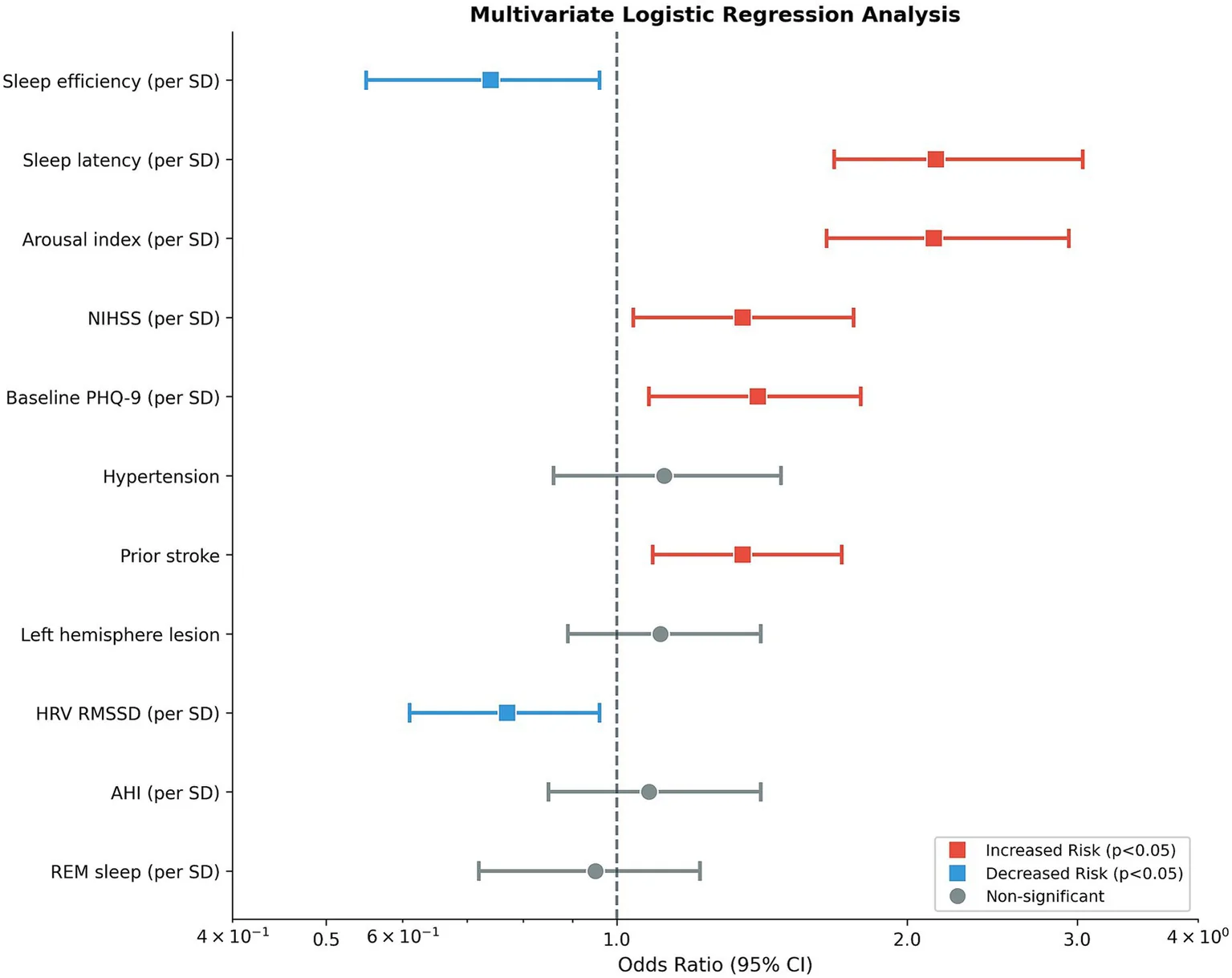
Forest plot of multivariate logistic regression analysis displaying odds ratios with 95% confidence intervals. Orange squares indicate significantly increased risk (*p* < 0.05); blue squares indicate significantly decreased risk; gray circles indicate non-significant associations. Dashed vertical line represents OR = 1.0 (no effect). CI, confidence interval; OR, odds ratio.

Figure 6 presents the forest plot of odds ratios from multivariate logistic regression analysis. After adjustment for potential confounders, several PSG parameters remained independently associated with PSD at 3-month follow-up. Reduced sleep efficiency was significantly associated with increased PSD risk (OR = 0.74 per standard deviation increase, 95% CI: 0.55–0.96, *p* = 0.026). Prolonged sleep latency demonstrated strong association with PSD (OR = 2.14 per standard deviation increase, 95% CI: 1.68–2.73, *p* < 0.001), as did elevated arousal index (OR = 2.13, 95% CI: 1.65–2.94, *p* < 0.001). Lower HRV RMSSD was independently associated with increased PSD risk (OR = 0.77 per standard deviation increase, 95% CI: 0.61–0.96, *p* = 0.020). Among clinical predictors, higher NIHSS score (OR = 1.35, 95% CI: 1.04–1.76, *p* = 0.024), higher baseline PHQ-9 score (OR = 1.40, 95% CI: 1.08–1.79, *p* = 0.010), and prior stroke history (OR = 1.35, 95% CI: 1.09–1.71, *p* = 0.004) were significant independent predictors. Neither discharge NIHSS, discharge mRS, nor receipt of reperfusion therapy retained independent significance after adjustment, and their inclusion did not materially alter the effect estimates for the sleep parameters. Hypertension, left hemisphere lesion, AHI, and REM sleep percentage did not achieve statistical significance in the multivariate model.

### Model performance and comparison

**Figure 7:**
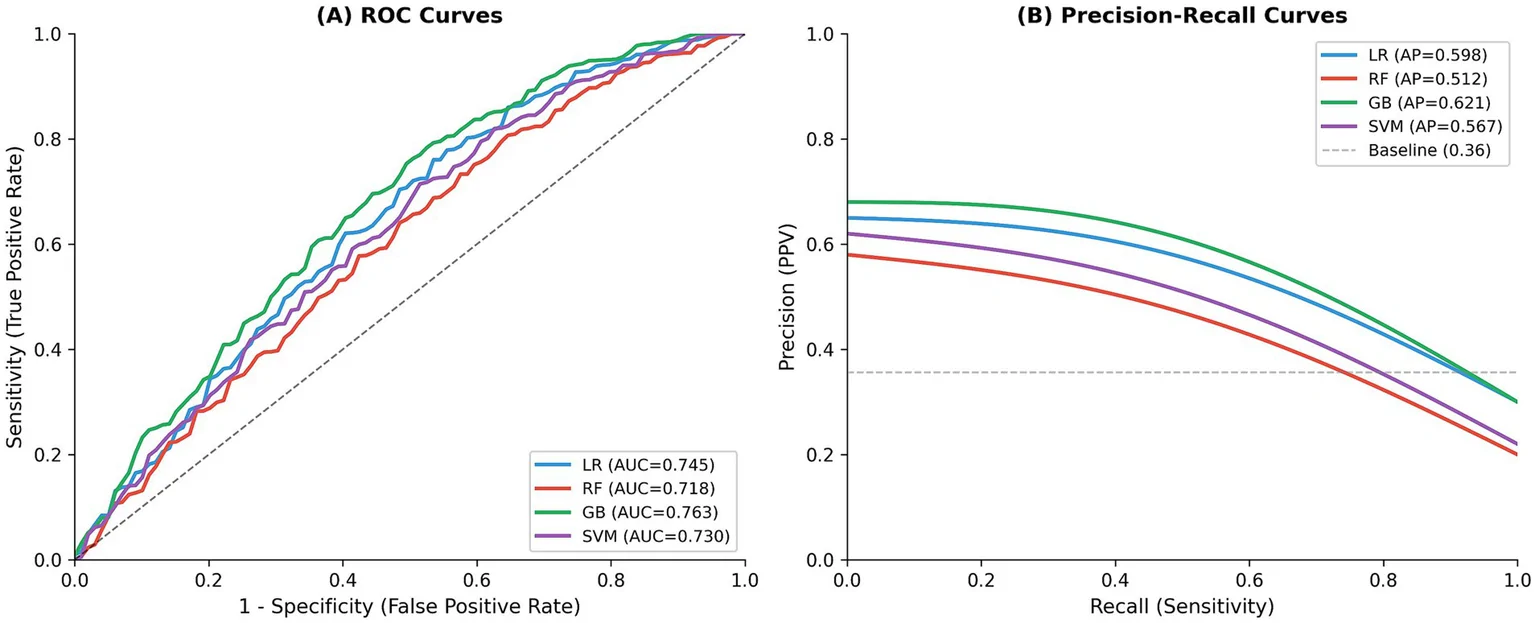
Model performance comparison. **(A)** Receiver operating characteristic (ROC) curves with area under the curve (AUC) values for each algorithm. **(B)** Precision-recall curves with average precision (AP) values. Dashed diagonal line in **(A)** represents random classifier performance. LR, logistic regression; RF, random forest; GB, gradient boosting; SVM, support vector machine.

Figure 7 illustrates the comparative performance of four prediction models. The receiver operating characteristic (ROC) curves (Figure 7A) demonstrated that all models achieved acceptable discrimination, with AUC values ranging from 0.718 to 0.763. Gradient boosting achieved the highest AUC (0.763), followed by logistic regression (0.745), SVM (0.730), and random forest (0.718). Precision-recall curves (Figure 7B) showed consistent patterns, with average precision values of 0.621 for gradient boosting, 0.598 for logistic regression, 0.567 for SVM, and 0.512 for random forest. At optimal thresholds determined by Youden’s index, logistic regression demonstrated sensitivity of 54.3% and specificity of 82.5%, while gradient boosting achieved sensitivity of 57.1% and specificity of 81.0%. Bootstrap internal validation confirmed model stability, with optimism-corrected AUC for logistic regression of 0.738 (95% CI: 0.682–0.794), representing appropriate optimism correction from the apparent AUC of 0.745.

### Feature importance analysis

**Figure 8:**
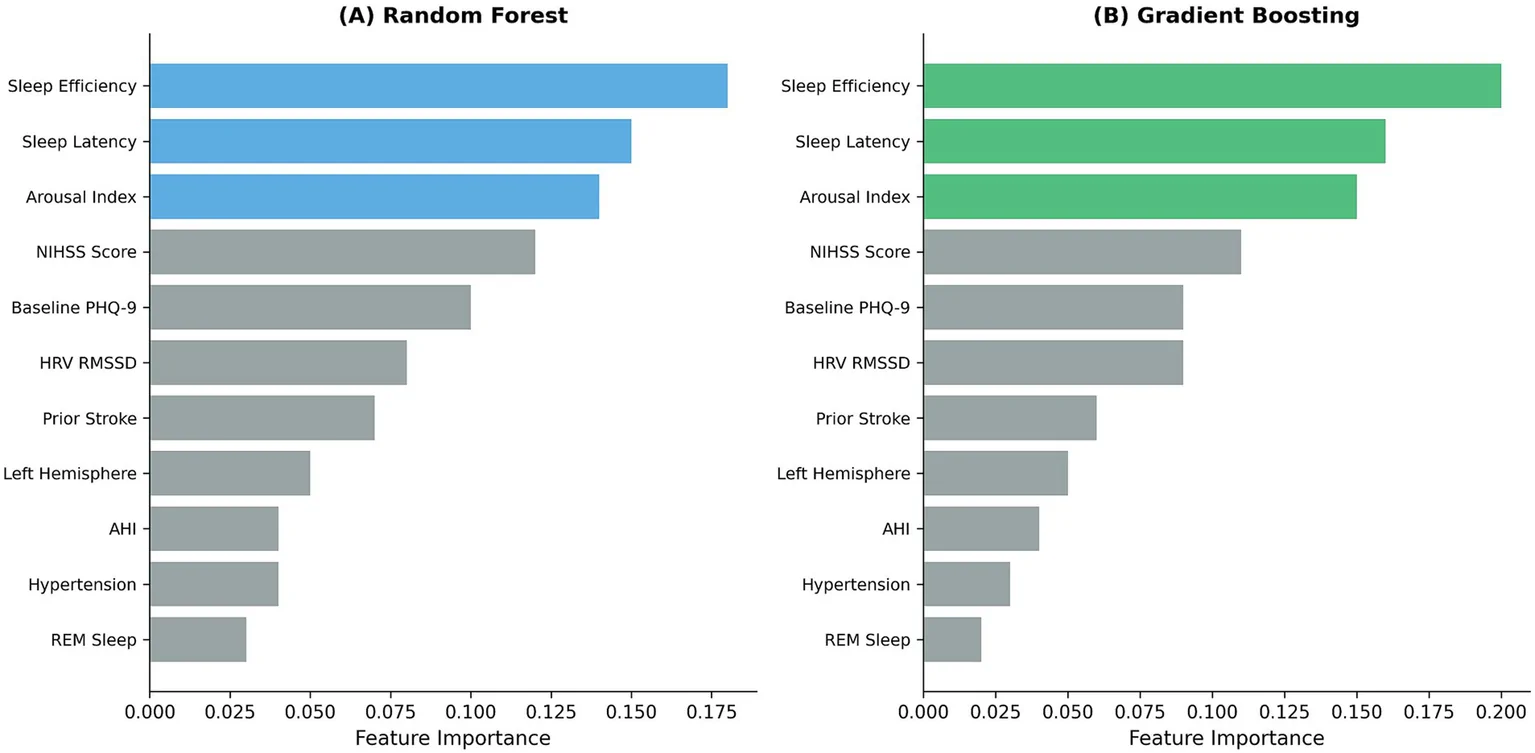
Feature importance analysis from ensemble machine learning models. **(A)** Random forest feature importance based on Gini impurity reduction. **(B)** Gradient boosting feature importance. Variables are ranked in descending order of importance. PSG, polysomnography; HRV, heart rate variability.

Feature importance rankings from ensemble methods are presented in Figure 8. Both random forest (Figure 8A) and gradient boosting (Figure 8B) algorithms consistently identified sleep efficiency as the most important predictor, followed by sleep latency and arousal index. This convergence across different algorithmic approaches strengthens confidence in the importance of these sleep architecture parameters for PSD prediction. NIHSS score and baseline PHQ-9 ranked among the top clinical predictors in both models. The consistency of feature rankings between methods suggests robust associations that are not artifacts of specific modeling approaches.

### Calibration and clinical utility

**Figure 9:**
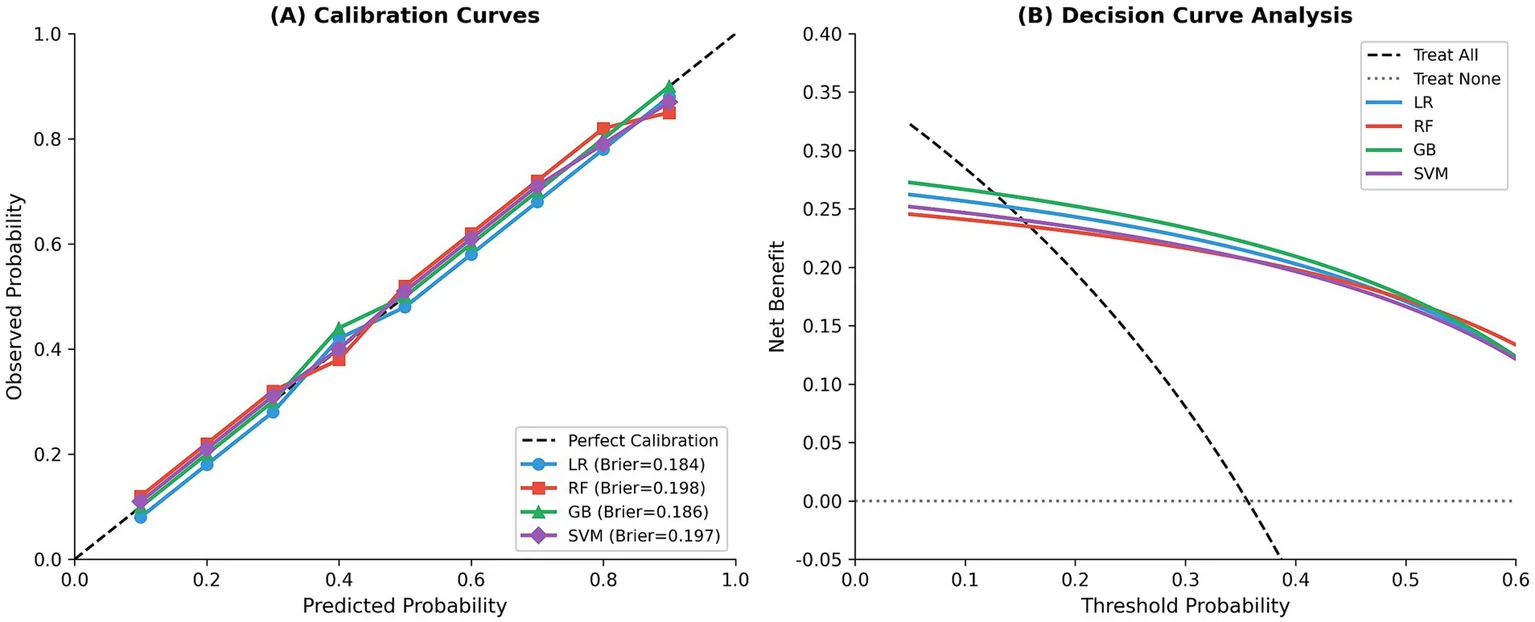
Model calibration and clinical utility assessment. **(A)** Calibration curves comparing predicted probabilities with observed event rates. Dashed diagonal line indicates perfect calibration. Brier scores are displayed for each model. **(B)** Decision curve analysis showing net benefit across threshold probabilities. Dashed line represents treat all strategy; dotted line represents treat none strategy.

Model calibration and clinical utility assessments are presented in Figure 9. Calibration curves (Figure 9A) demonstrated that logistic regression and random forest models tracked closely with the diagonal reference line indicating perfect calibration, with Brier scores of 0.184 and 0.198, respectively. Gradient boosting showed slightly inferior calibration (Brier score 0.186) despite achieving the highest discrimination. Decision curve analysis (Figure 9B) evaluated clinical utility across a range of threshold probabilities. All models provided positive net benefit compared to default strategies (treat all or treat none) across threshold probabilities from approximately 10 to 50%, indicating clinical utility for decision-making within this clinically relevant range.

### Risk stratification

**Figure 10:**
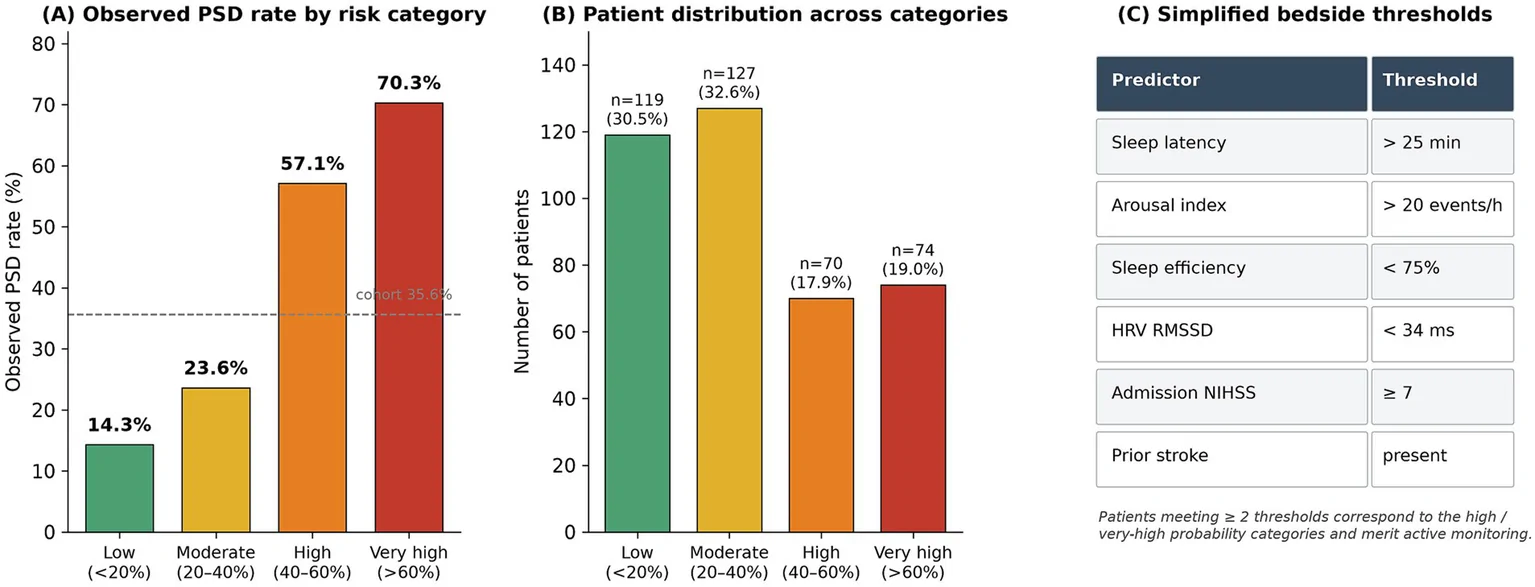
Risk stratification and clinical decision support. **(A)** Observed PSD rate across the four predicted-probability categories; The dashed line marks the overall cohort rate (35.6%). **(B)** Distribution of patients across the four risk categories. **(C)** Simplified bedside thresholds for the principal predictors to support clinical decision-making. PSD, post-stroke depression; NIHSS, National Institutes of Health Stroke Scale; HRV RMSSD, heart rate variability root mean square of successive differences.

Figure 10 presents the risk stratification analysis and clinical decision support summary. Using predicted probability cutoffs, patients were classified into four risk categories: low risk (less than 20%, *n* = 119, 30.5%), moderate risk (20–40%, *n* = 127, 32.6%), high risk (40–60%, *n* = 70, 17.9%), and very high risk (greater than 60%, *n* = 74, 19.0%). Observed PSD rates demonstrated excellent discrimination across categories: 14.3% in low-risk, 23.6% in moderate-risk, 57.1% in high-risk, and 70.3% in very high-risk groups. To facilitate bedside application, simple parameter thresholds corresponding to the upper-risk range were derived: sleep latency greater than 25 min, arousal index greater than 20 events per hour, sleep efficiency below 75%, and HRV RMSSD below 34 ms; the four-category probability score, together with these thresholds, is summarized in Figure 10 to support clinical decision-making. The calibration plot showed good agreement between predicted probabilities and observed rates across deciles. The top predictors summary provides a practical reference for clinical decision-making, highlighting sleep efficiency, sleep latency, and arousal index as the most influential PSG-derived predictors.

## Discussion

This prospective cohort study establishes that PSG parameters, particularly sleep efficiency, sleep latency, and arousal index, constitute significant independent predictors of PSD at 3-month follow-up in acute ischemic stroke patients. The prediction model incorporating both clinical characteristics and PSG parameters achieved good discriminative ability with AUC values exceeding 0.74 across multiple modeling approaches. These findings align with recent meta-analytic evidence reporting pooled c-indices ranging from 0.72 to 0.82 for machine learning-based PSD prediction models (Wu et al., 2025), while extending prior work through the systematic incorporation of comprehensive sleep architecture parameters. The gradient boosting model demonstrated the highest discriminative performance (AUC = 0.763), consistent with emerging evidence supporting ensemble methods for clinical prediction tasks in stroke populations (Gu et al., 2025; Zuo and Yang, 2025).

The observed PSD incidence of 35.6% at 3-month follow-up in our cohort demonstrates consistency with published literature documenting prevalence rates of 25–35% during the first year following stroke (Hackett and Pickles, 2014; Robinson and Jorge, 2016; Liu et al., 2023). A comprehensive systematic review by Liu and colleagues reported that approximately 71% of incident depression cases during the first year post-stroke have onset within 3 months, underscoring the importance of early risk identification during this critical period (Liu et al., 2023). Our finding that prolonged sleep latency (OR = 2.14) and elevated arousal index (OR = 2.13) were among the strongest predictors of PSD aligns with growing evidence from polysomnographic studies linking sleep fragmentation and difficulty initiating sleep with depressive disorders in both stroke and non-stroke populations (Fang et al., 2019; Yasugaki et al., 2025). The mechanistic relationship likely involves bidirectional interactions wherein acute stroke disrupts sleep-regulating neural circuits, and the resulting sleep disturbances subsequently promote inflammatory processes, hypothalamic–pituitary–adrenal axis dysregulation, and neuroplasticity impairments that collectively increase depression vulnerability (Miller et al., 2009; Villa et al., 2018).

The protective effect of higher sleep efficiency (OR = 0.74) on PSD development merits particular attention given its clinical interpretability and potential for intervention. Sleep efficiency, calculated as the ratio of total sleep time to time in bed, provides an integrated measure of sleep initiation and maintenance capacity that captures multiple pathophysiological processes relevant to mood regulation. Previous cross-sectional investigations have reported associations between reduced sleep efficiency and depressive symptoms in stroke patients, but the prospective design of the current study provides stronger evidence for a temporal relationship supporting sleep efficiency as a predictive biomarker rather than merely a correlate of established depression (Wang et al., 2025). Polysomnographic studies in non-stroke depressed populations have consistently demonstrated reduced sleep efficiency, prolonged sleep latency, and increased arousal frequency as characteristic features of the depressive sleep phenotype (Baglioni et al., 2011; Alvaro et al., 2013; Benca et al., 1992).

The significant association between reduced HRV RMSSD and increased PSD risk (OR = 0.77) contributes important evidence regarding autonomic mechanisms in PSD pathophysiology. RMSSD primarily reflects cardiac parasympathetic modulation, and its reduction indicates diminished vagal tone that may predispose to depression through multiple pathways (Sgoifo et al., 2015; Koch et al., 2019). Meta-analytic evidence has established that patients with major depressive disorder demonstrate significantly reduced HRV compared to healthy controls, with effect sizes ranging from moderate to large depending on the specific HRV index examined (Kemp et al., 2010; Alvares et al., 2016). The vagus nerve has emerged as a critical component of the inflammatory reflex, whereby parasympathetic activity suppresses systemic inflammation through the cholinergic anti-inflammatory pathway. Reduced vagal tone, as reflected by lower HRV, may therefore contribute to the pro-inflammatory state that has been implicated in PSD pathogenesis (Miller et al., 2009; Zhou et al., 2025). Furthermore, the polyvagal theory proposed by Porges suggests that vagal regulation plays a fundamental role in emotional regulation and social engagement, providing theoretical support for the HRV-depression relationship observed in our study (Porges, 2009).

Among clinical predictors, NIHSS score demonstrated independent predictive value for PSD (OR = 1.35), consistent with extensive prior literature establishing stroke severity as a robust predictor across diverse populations and assessment timepoints (Towfighi et al., 2017; Ayerbe et al., 2013; Medeiros et al., 2020). Higher baseline PHQ-9 score was independently associated with PSD development (OR = 1.40), emphasizing the importance of subclinical depressive symptoms as risk markers even when they do not meet diagnostic threshold criteria. This finding aligns with meta-analytic evidence identifying previous depression history and early depressive symptoms as among the strongest predictors of subsequent PSD, with relative risks ranging from 1.5 to 2.3 (Taylor-Rowan et al., 2019; Mitchell et al., 2017). Prior stroke history emerged as an independent predictor (OR = 1.35), suggesting that cumulative cerebrovascular burden may increase depression vulnerability through mechanisms that may include greater extent of subclinical white matter disease, reduced cognitive reserve, or psychosocial factors related to recurrent illness experience (Ferro et al., 2016; Tang et al., 2011; Ríos et al., 2025).

Although lesion topography was highlighted as a leading hypothesis in the introduction, neither left-hemisphere involvement nor frontal–subcortical location was independently associated with PSD in the present cohort (Figure 2F). Several factors may reconcile this observation with the lesion-location literature, including the heterogeneity of infarct distribution in an unselected acute cohort, the modest proportion of strategically located infarcts, and the possibility that dynamic sleep and autonomic markers capture downstream network dysfunction more sensitively than anatomical location alone. This finding does not refute the lesion-location hypothesis but indicates that, at the cohort level, physiological sleep parameters carried greater predictive weight than lesion topography.

The feature importance analysis revealed consistent rankings across random forest and gradient boosting algorithms, with sleep efficiency, sleep latency, and arousal index emerging as the most influential predictors. This methodological triangulation strengthens confidence that the identified associations reflect genuine biological relationships rather than artifacts of specific modeling assumptions. The convergence of traditional statistical analysis (multivariate logistic regression) and machine learning approaches provides complementary evidence supporting the importance of sleep architecture parameters in PSD prediction. Decision curve analysis demonstrated that the developed models provide clinical utility across threshold probabilities relevant to clinical decision-making (10–50%), suggesting potential value in guiding resource allocation for preventive interventions (Vickers and Elkin, 2006).

### Limitations

Several limitations warrant consideration when interpreting these findings. First, the single-center design may limit generalizability to populations with different demographic characteristics, healthcare systems, or stroke care protocols. External validation in independent cohorts will be essential to establish the transportability of our prediction model. Second, while we adjusted for multiple potential confounders, unmeasured factors including genetic susceptibility and detailed psychosocial variables may have influenced our results. Although no participant was receiving hypnotic therapy at baseline and concomitant psychotropic use was recorded, a proportion of patients initiated mood-relevant medication during follow-up (antidepressants 9.5%, hypnotics 6.4%), which may have influenced symptom trajectories despite adjustment. Third, PSG was performed within 7 days of stroke onset, and sleep patterns may continue to evolve during the recovery period; serial PSG assessments might capture dynamic changes with additional predictive value. Fourth, we utilized PHQ-9 for outcome assessment rather than structured clinical interviews, though PHQ-9 has demonstrated good psychometric properties for depression screening in stroke populations with sensitivity and specificity exceeding 80% at the threshold of 10 (Kroenke et al., 2001; Williams et al., 2005); consequently, some cases identified by a PHQ-9 score ≥10 may not meet formal DSM-5 criteria for major depressive disorder, and case ascertainment should be interpreted with this in mind. Fifth, the moderate sample size, while adequate for the events per variable ratio in our primary analysis, limited our ability to examine interactions between predictors or to develop more complex machine learning architectures that might improve performance.

In conclusion, PSG parameters, particularly sleep efficiency, sleep latency, and arousal index, constitute significant independent predictors of PSD in acute ischemic stroke patients. The integration of objective sleep assessment into early stroke management protocols may facilitate identification of high-risk patients and enable targeted implementation of preventive interventions. Future directions should include external validation studies, investigation of whether sleep-focused interventions can reduce PSD incidence in high-risk patients identified through PSG screening, and exploration of the neurobiological mechanisms linking sleep disturbance to depression vulnerability in the post-stroke period. The development of simplified screening tools that capture the most predictive sleep parameters may facilitate broader clinical implementation of risk stratification approaches.

## Data Availability

The raw data supporting the conclusions of this article will be made available by the authors, without undue reservation.
